# Characterization of neuropeptides which control cerebellar granule cell survival, migration and differentiation

**DOI:** 10.1186/2193-1801-4-S1-L58

**Published:** 2015-06-12

**Authors:** David Vaudry, Auriane Corbière, Magalie Basille, Seyma Bahdoudi, Olfa Masmoudi, Jérome Leprince, Delphine Burel, Magalie Bénard, Ludovic Galas, France Hitoshi Komuro

**Affiliations:** INSERM U982, Rouen University, France; Tunis University, Tunisia; PRIMACEN, INSERM, France; Yale University, New Haven, USA

**Keywords:** cerebellar granule neurons, neuropeptides, brain development

During cerebellar development, granule cell precursors are produced from a secondary germinative zone forming the external granule cell layer (EGL). Immature granule neurons from the inner part of the EGL then start a tangential migration followed by a centripetal inward radial migration across the molecular and Purkinje cell layers to reach their final destination at the bottom of the forming internal granule cell layer (IGL). This complex migratory process is highly regulated and takes about 2 days in rodents and it is essential for the proper formation of the cortical layers forming the mature cerebellum. In the IGL, granule cells differentiate to establish functional excitatory synapses with GABAergic neurons including Purkinje, basket, stellate and Golgi cells, or die. Some neurotrophins and neurotransmitters have been shown to be involved in the control of cerebellar granule cell survival, migration and differentiation. Initially, when I started my carrier as a researcher, we used to claim that very few neuropeptides were produced in the cerebellum. Nevertheless, we now know that this was wrong as we have recently identified by mass spectrometry over 70 peptides expressed in the cerebellum during development. Over the years, the involvement of some of these peptides such as somatostatin, PACAP or ODN, has been established in the control of cerebellar granule cell survival, migration and differentiation as will illustrate my presentation.

